# Supplementary material for: A social network analysis of fraud prediction on crowdsourcing platforms
Source: PLoS One. 2026 Mar 11;21(3):e0343412. doi: 10.1371/journal.pone.0343412 (PMC12978447; doi:10.1371/journal.pone.0343412)
Supplement: Attachment — Submitted filename: Response to reviewers.docx [file pone.0343412.s002.docx]

# Response to Reviewers

We would like to express our sincere gratitude for your thorough review and thoughtful feedback on our manuscript. Your constructive comments and suggestions have greatly contributed to enhancing the quality and clarity of our work. We have carefully considered all the reviewer comments and revised the manuscript in response.

In summary, the following key areas have been addressed in the revised version, with the changes highlighted in yellow for your convenience:

1. **Enhanced Analytical Methods and Rigor**

- Supplemented Quantitative Analysis: Added effect size analyses (e.g., Cohen’s d, CLES) for key findings to complement statistical significance tests and assess the practical magnitude of observed effects.
- Conducted Sensitivity Tests: Performed and reported systematic sensitivity analyses on critical network construction parameters (time window, edge-weighting). This confirms the robustness of our core conclusions across a plausible range of values, with results presented in new tables.
- Highlighted Model Validation: Further clarified the use of comprehensive model evaluation metrics and emphasized the McNemar's test to statistically validate the significant performance gain contributed by the novel social network features.
- Strengthened Methodological Justification: Expanded the rationale in the "Construct the interaction structure from relationships" section, providing theoretical and empirical grounding for the 3 - month time window and the edge - weighting scheme.

2. **Clarification of Key Concepts and Terminology**

- Explicit Definitions and Process: Added a clear definition of fraudulent users and a detailed, step - by - step description of the platform's three - step, ex - post identification and verification process (reporting, investigation, announcement) in the Introduction and Data Description sections.
- Consistent Terminology: Implemented a context - based terminology strategy throughout the manuscript (e.g., "fraudulent (task) seekers," "fraudulent nodes," "fraudsters") to ensure precise and consistent expression across different sections (e.g., Introduction, Methodology, Discussion).

3. **Refined Presentation of Content and Conclusions**

- Calibrated Conclusions: Toned down overly absolute claims and removed unsupported speculative interpretations (e.g., regarding fraudsters’ "intentions" or "strategies"), ensuring all inferences are strictly grounded in the empirical data.
- Focused, Objective Interpretation: In the Discussion, reframed explanations to focus on observable behavioral patterns and the functional implications of network positions, avoiding unsubstantiated psychological conjectures.

4. **Optimized Language and Structure**

- Comprehensive Language Polishing: Revised the manuscript to correct grammatical and phrasing issues and had it polished by a native English-speaking academic in the field to enhance fluency and clarity.
- Streamlined Content: Eliminated repetitive content, condensed overly long sections, and improved the logical flow and structure of several sections to increase readability.

5. **Updated Limitations and Compliance Statements**

- Acknowledged Study Limitations: Added explicit discussions on the potential impact of non - independent observations (due to seekers appearing in multiple projects) and the use of down - sampling for class imbalance as part of the study's limitations.
- Ensured Policy Compliance: Revised the Data Availability Statement in accordance with journal policy and confirmed the funders' non - involvement in the study.
- These systematic revisions aim to significantly strengthen the manuscript's methodological rigor, clarity of presentation, and reliability of conclusions. Specific point - by - point details are provided in the subsequent responses to each reviewer.

**Response to the editor**

*I will require the authors to add a clear definition (and brief description of the identification/labeling process) distinguishing fraudulent vs non-fraudulent users, and to ensure terminology is used consistently throughout the manuscript.*

**Response:**

Thank you for your valuable feedback. We have thoroughly revised the paper in response to your comments, as detailed below.

1. **Clear Definition and Identification/Labeling Process**

As suggested, we have added a clear definition and description of the process used to distinguish fraudulent from non-fraudulent users, which are detailed in Section Introduction (pp.1-2) and Section Data Source and Data Description (p.10). The platform identifies fraudulent seekers through a three-step, ex-post process:

- **Reporting and Evidence Collection:** The process is initiated when solvers submit reports or feedback alleging fraudulent activities by a seeker (e.g., solution theft, payment refusal, double identity fraud).
- **Manual Investigation and Verification:** The platform administration investigates the allegations based on the submitted reports and evidence. This may involve comparing solutions, analyzing user IDs, and reviewing transaction records.
- **Judgment and Announcement:** Based on the investigation, a determination of fraud is made and verified fraud cases are publicly announced on the platform’s bulletin board.

In this study, we leveraged the outcome of this process. The publicly listed fraudulent seekers from the platform’s official announcements were directly used to label and identify fraudulent accounts in our dataset.

2. **Ensuring Terminological Consistency Throughout the Manuscript**

We have systematically revised the manuscript’s terminology to ensure rigorous consistency, adopting a context-based application strategy:

- **For the platform user behavior context** of this study (e.g., Introduction), we used fraudulent (task) seekers and legitimate/non-fraudulent seekers（users）, aligning with real-world crowdsourcing scenarios for intuitive comprehension.
- **For the social network structural analysis context** (e.g., Methodology), we used fraudulent nodes and non-fraudulent nodes to ensure analytical precision; a transition paragraph in the Methodology chapter clarifies the conversion from real-world seekers to network nodes.
- **When referring to fraudulent actors in other generic fraud scenarios** (beyond the study’s crowdsourcing platform context), we uniformly used the term fraudster for consistent expression.
- **In cross-context sections** (e.g., Discussion), we appropriately integrated the above term sets based on contextual logic, with all terms consistently referring to the same research entities and no ambiguity.

All relevant revisions have been implemented across the full manuscript in accordance with the above principles. Given the fragmented nature of the terminological revisions scattered throughout the text, only the key additions/revisions in Section Introduction and Section Data source and data description (definition and identification process) have been highlighted; the scattered terminological adjustments across other sections follow the aforementioned strategy strictly. We sincerely appreciate your insightful comments.

3. **Role of Funder Statement:**

This work was supported by the Natural Science Foundation of Sichuan Province, China (Grant No. 24NSFSC3739) and the Ministry of Education Humanities and Social Sciences, China (Grant No. 24YJC630301). The funders played no role in study design, data collection and analysis, decision to publish, or preparation of the manuscript.

4. **Revised Data Availability Statement**

The datasets were collected under confidentiality restrictions from a leading crowdsourcing platform in China and are used under license for this study, thus cannot be shared via public repositories. To ensure long-term availability per journal policy, access is managed through two contacts: 1)Wenjie Zhang(wenjie_zhang@swufe.edu.cn); 2) Independent data steward (non-author) Liting Li at Taiyuan University of Technology (liliting@tyut.edu.cn). Data can be accessed upon reasonable request, subject to platform permission. The data stewards commit to preserving the data and responding to requests for at least 10 years.

**Response to the Reviewer 1**

*Excellent clarity throughout the work. Strong organization and flow. Ideas are presented with confidence. Very effective use of supporting details. The writing remains focused and purposeful. Insightful analysis that adds real value. The argumentation is compelling and smooth. Demonstrates impressive subject knowledge. Thoughtful structure enhances readability.*

**Response：**

Thank you for your positive evaluation of our manuscript. We greatly appreciate your support and the trust you placed in our work. Based on your encouraging comments, we carefully reviewed our manuscript and implemented several stylistic and structural improvements to enhance clarity and academic rigor.

**Response to the Reviewer 2**

*The study addresses an important topic and applies social network analysis and machine-learning methods to fraud prediction on crowdsourcing platforms. While the dataset is strong and the research direction is valuable, the manuscript requires substantial revision before it can be considered for publication.*

**Response：**

Thank you for your valuable feedback. We have thoroughly revised the paper in response to your comments, as detailed below.

1. **The statistical methods used are appropriate in principle, but the analysis lacks rigor—effect sizes, diagnostics, sensitivity tests, and full model evaluation metrics are missing.**
    Thank you for highlighting the importance of methodological rigor. In response to your comment, we have significantly strengthened our analysis and clarified our validation approach across multiple dimensions. Our revisions and clarifications are detailed below.
(1) **Provision of Full Model Evaluation Metrics and Diagnostics**

We wish to clarify that comprehensive model evaluation metrics were already included in the original manuscript. Table 10 (The Model Performance Evaluation Results) reports key performance indicators—Accuracy, Precision, Recall, F1 Score, and AUC—for both baseline models (BM1, BM2) and our social network-augmented models (SN-BM1, SN-BM2).

Crucially, to statistically test the incremental value of the newly added social network features, we had already performed and reported a McNemar's test. This paired test formally compares classifier performance. The results (p-values of 0.0352 and 0.0221) provide direct statistical evidence that the performance improvement gained by adding centrality and cohesion features is significant.

Regarding diagnostics, the core objective of our modeling was to validate the incremental predictive contribution of our novel social network constructs to established baselines. Therefore, our diagnostic focus was appropriately placed on validating this addition through: (a) cross-validated performance comparison (Table 10), (b) formal statistical testing of the improvement (McNemar's test), and (c) examination of feature importance rankings from the Random Forest, which confirmed the relevance of the new network metrics.

(2) **Addition of Effect Size Analysis**

To move beyond reliance on statistical significance alone and to assess the practical significance of our findings, we have now added standardized effect size reporting: In Section Analysis of centrality and cohesion (pp.14-15), we have calculated and reported Cohen’s d values for key comparisons involving centrality metrics; In Section Analysis of structural equivalence (pp.15-16), we have supplemented the structural equivalence analysis with the Common Language Effect Size (CLES) for key findings. These measures provide clear, interpretable metrics of the substantive magnitude of the observed differences.

(3) **Conduct of Sensitivity Tests**

To address potential concerns about the arbitrariness of key parameters in our network construction, we have conducted and reported a full sensitivity analysis (pp.16-17). We systematically varied the communication edge-weight coefficient and the aggregation time window across a plausible range of values. While the statistical significance level remains at p < 0.001 or p < 0.01 for the vast majority of tests and shows only minimal relaxation under the most extreme parameter settings, the direction and substantive interpretation of all results are entirely unchanged. This demonstrates that our key conclusions are not driven by specific methodological choices but are reliable properties of the data.

In summary, we have addressed the concerns regarding rigor by: (a) clarifying and highlighting our comprehensive model evaluation and incremental validation strategy (including McNemar's test), (b) supplementing the analysis with effect sizes, and (c) demonstrating the robustness of our conclusions through extensive sensitivity testing. We believe these efforts collectively have provided a rigorous, multi-faceted validation of our study's findings. We thank you for your insightful comments, which have greatly improved the clarity and strength of our manuscript.

2. **Several conclusions are overstated or speculative relative to the data.**

Thank you for this valuable comment. We acknowledge that some conclusions were overstated or speculative, and have thoroughly revised the manuscript to ensure all inferences are strictly grounded in empirical data. We softened absolute language to align with our findings, removed all subjective interpretations unsupported by data (such as inferences about fraudsters’ "intentions" or "strategies"), and explicitly clarified the boundary of our conclusions — emphasizing they are specific to our task-oriented crowdsourcing context rather than generalizable to all fraud types or platforms. Additionally, we strengthened the link between data and conclusions by referencing corresponding statistical results for each key claim, ensuring our findings are proportionate to the evidence and enhancing the manuscript’s objectivity and rigor.

3. **The manuscript also requires major improvements in clarity, structure, and English writing; key sections are repetitive, overly long, and contain grammatical and phrasing issues that hinder understanding.**

Thank you for your valuable comments on the manuscript’s clarity, structure and English writing. We fully acknowledge these issues and have conducted a comprehensive section-by-section revision to address them thoroughly: we eliminated repetitive content and streamlined overly long passages in key sections, rectified all identified grammatical and phrasing errors that could hinder understanding, optimized the logical structure and coherence of the whole manuscript by adjusting subsection order and adding appropriate transitional expressions, and standardized the use of professional terminology for consistent expression. Additionally, the entire manuscript has been polished by a native English-speaking academic with expertise in our research field to further enhance linguistic accuracy and fluency. All the above revisions are clearly marked in the revised manuscript for your easy review.

4. **Methodological explanations (e.g., window size, edge weighting, fraud labeling) must be clearer and better justified.**

Thank you for your valuable comments. We have revised the manuscript accordingly to provide clearer and better-justified explanations for our key methodological choices. The specific modifications and additions are detailed below.

(1) **Regarding the Fraud Labeling Process**

The platform identifies fraudulent seekers through a three-step, ex-post process:

- Reporting and Evidence Collection: The process is initiated when solvers submit reports or feedback alleging fraudulent activity by a seeker (e.g., solution theft, payment refusal, double identity fraud).
- Manual Investigation and Verification: The platform administration investigates the allegations based on the submitted reports and evidence. This may involve comparing solutions, analyzing user IDs, and reviewing transaction records.
- Judgment and Announcement: Based on the investigation, a determination of fraud is made and verified fraud cases are publicly announced on the platform’s bulletin board.

We also explicitly link the platform’s official fraud labels to our dataset annotation to further confirm the validity of the dependent variable.

(2) **Regarding the Justification for the Time Window and Edge-Weighting Scheme**

We have significantly expanded the discussion in Section Construct the interaction structure from relationships (pp.10-11) to provide a clear, multi - faceted rationale for the 3 - month time window and the edge weighting scheme. The three-month sliding window is adopted based on theoretical, practical, and methodological considerations. Theoretically, it aligns with the sociological understanding that tie strength decays without recent contact. Practically, it corresponds to the typical task lifecycle on the platform, ensuring the network reflects relevant, ongoing collaborations. Methodologically, it balances dynamism and stability by avoiding the sparse, volatile structures of shorter windows and preventing the inclusion of obsolete connections from longer windows, thereby preserving the network's contemporaneity.

The edge-weighting scheme is similarly grounded in empirical observations of collaboration intensity on the platform. It is designed to more precisely capture the varying influence of nodal relationships rather than relying on simple binary connections. The detailed rationale for this scheme has also been added to Section Construct the interaction structure from relationships (pp.10-11).

More importantly, to directly address the concern about arbitrariness and demonstrate the stability of our findings, we have conducted and reported a comprehensive sensitivity analysis (pp.16-17).

5. **The discussion should more carefully interpret findings and avoid unsupported psychological explanations.**

Thank you for this valuable suggestion. In the revised Discussion section (pp.21-22), we have systematically reframed our interpretation to avoid any speculation about psychological intent. Our explanations now focus squarely on observable behavioral patterns (e.g., "direct, dyadic interactions") and the functional implications of network positions (e.g., "a peripheral location inherently reduces visibility"). We explicitly anchor the analysis in the objective context of the platform ecology (e.g., task-oriented, project-based cycles) and the specific fraud objective (solution plagiarism), showing how these factors shape the distinct structural signatures we identified. This ensures our interpretation remains grounded in the empirical data and network logic, enhancing the section's objectivity and rigor.

**Response to the Reviewer 3**

*Thank you for the opportunity to review this manuscript. The topic is timely and relevant, and the authors address an important issue in crowdsourcing platforms by examining how social network features can help identify fraudulent seekers. Overall, the manuscript is clear, well organized, and generally easy to follow. Despite these points, the overall methodology is coherent, and the main findings—that fraudulent seekers tend to occupy less-connected positions in the network and that SNA features improve prediction accuracy—are supported by the data.*

**Response：**

Thank you for your insightful and constructive comments, as well as your positive evaluation of our manuscript’s topic relevance, technical soundness, methodological coherence and practical contributions. We greatly appreciate your recognition of the dynamic social network construction, statistical methods and fraud-prediction experiment, and we have carefully addressed all the raised concerns and fully adopted your suggestions for improvement. The detailed revisions are presented below by key themes:

1. **Platform Fraud Identification and Validation Process Clarification**

We acknowledge your comment that the platform’s fraud identification process was insufficiently described, and this process is central to the credibility of our fraud labels. As suggested, we have added a detailed, step-by-step explanation of how the platform identifies and validates fraudulent seekers in Section Data Source and data description (p.10). We also explicitly link the platform’s official fraud labels to our dataset annotation to further confirm the validity of the dependent variable.

The platform identifies fraudulent seekers through a three-step, ex-post process:

- **Reporting and Evidence Collection:** The process is initiated when solvers submit reports or feedback alleging fraudulent activity by a seeker (e.g., solution theft, payment refusal, double identity fraud).
- **Manual Investigation and Verification:** The platform administration investigates the allegations based on the submitted reports and evidence. This may involve comparing solutions, analyzing user IDs, and reviewing transaction records.
- **Judgment and Announcement:** Based on the investigation, a determination of fraud is made and verified fraud cases are publicly announced on the platform’s bulletin board.

2. **Discussion of Non-independent Observations from Repeated Seekers**

We agree that the non-independence of observations caused by seekers appearing in multiple projects may affect statistical significance levels. Following your suggestion, we have added a dedicated discussion of this limitation in Section Conclusion and Limitations (pp.22-23); we specifically analyze its potential impact on our statistical tests and emphasize that we have interpreted the results with full consideration of this issue, and supplemented the Common Language Effect Size (CLES) of key findings in Section Analysis of structural equivalence (pp.15-16) to avoid over-reliance on statistical significance alone.

3. **Justification of Key Assumptions for Network Construction**

Thank you for raising this important point regarding the justification of our key methodological choices. In direct response to your comment, we have significantly expanded the discussion in Section Construct the interaction structure from relationships (pp.10-11) to provide a clear, multi - faceted rationale for the 3 - month time window and the edge weighting scheme.

First, we now explicitly justify these choices based on theoretical reasoning and platform - specific empirical patterns (e.g., typical project lifecycles and interaction decay). More importantly, to directly address the concern about arbitrariness and demonstrate the stability of our findings, we have conducted and reported a comprehensive sensitivity analysis (pp.16-17).

This analysis formally tests the robustness of our core conclusions by varying the communication edge weight coefficient across {0.3, 0.5 (baseline), 0.7} and the time window across {2, 3 (baseline), 4} months. The results, summarized in the new Tables 6 and 7. The core descriptive pattern holds universally across all parameter combinations. While the statistical significance level remains at p < 0.001 or p < 0.01 for the vast majority of tests and shows only minimal relaxation under the most extreme parameter settings, the direction and substantive interpretation of all results are entirely unchanged. This demonstrates that our key conclusions are not driven by specific methodological choices but are reliable properties of the data.

Therefore, we demonstrate that our primary findings are not artifacts of the specific baseline parameters but are robust across a plausible range of alternative values. This substantive addition strengthens the methodological foundation of our study and directly validates the assumptions questioned in your review. We believe this satisfactorily mitigates the concern about arbitrariness.

4. **Interpretation of the Small Numerical Values for Centrality Metrics**

We thank the reviewer for raising this important point. We fully agree that the absolute magnitude of normalized centrality measures in large-scale networks requires careful contextual interpretation.

In response, we have made the following revisions to the manuscript:

- Added Explanatory Text: In Section Descriptive statistics (p.14), we have added a paragraph clarifying that the small absolute values of centrality metrics (e.g., degree centrality on the order of 0.001) are an expected outcome in large, sparse networks like ours, where normalization factors are immense. We explicitly guide readers to focus on the relative differences and statistical associations rather than the absolute numbers.
- Supplemented with Effect Size Analysis: Most importantly, to directly address the question of “how meaningful are these differences?”, we have now calculated and reported Cohen’s d values for key comparisons involving centrality metrics (e.g., fraudulent vs. non-fraudulent seekers) in Section Analysis of Centrality and Cohesion (pp.14-15). Reporting this standardized effect size provides readers with a clear, interpretable metric of practical significance, complementing the statistical significance tests.

These additions ensure that readers can accurately assess the importance of network positioning in our study, even when the raw centrality coefficients are numerically small.

5. **Acknowledgment of Down-sampling Limitation for Imbalanced Data**

We recognize your concern that down-sampling the non-fraud group may reduce dataset representativeness. We have added an explicit acknowledgment of this limitation in Section Conclusion and Limitations (pp.22-23), and briefly discuss alternative imbalance-handling strategies (e.g., SMOTE, weighted loss functions) that could be used in future research to complement our findings; we also note that the cross-validation we adopted has mitigated the potential bias of down-sampling to a certain extent.

6. **Revision of Data Availability Statement to Comply with PLOS ONE Policies**

Thank you for the reminder. We have revised the Data Availability Statement in strict compliance with PLOS ONE’s relevant policies, and the updated statement has been incorporated into the revised manuscript as required.
